# One-step synthesis of M13 phage-based nanoparticles and their fluorescence properties

**DOI:** 10.1039/d0ra02835e

**Published:** 2021-01-05

**Authors:** Jing Yi Lai, Naoya Inoue, Chuan Wei Oo, Hideya Kawasaki, Theam Soon Lim

**Affiliations:** Institute for Research in Molecular Medicine, Universiti Sains Malaysia 11800 Penang Malaysia theamsoon@usm.my +60-4-653-4803 +60-4-653-4852; Department of Chemistry and Materials Engineering, Faculty of Chemistry, Materials and Bioengineering, Kansai University 3-3-35 Yamate-cho Suita-shi Osaka 564-8680 Japan hkawa@kansai-u.ac.jp +60-6-6368-0979; School of Chemical Sciences, Universiti Sains Malaysia 11800 Minden Penang Malaysia; Analytical Biochemistry Research Centre, Universiti Sains Malaysia 11800 Penang Malaysia

## Abstract

Fluorescent carbon nanoparticles have been gaining more attention in recent years for their excellent fluorescence properties and simple synthesis routes. Different carbon sources have been reported for fluorescent carbon nanoparticle synthesis but the use of virus particles as a carbon source is scarce. Herein, we report the utilization of M13 bacteriophage particles as the carbon source to synthesize phage-based nanoparticles through facile, one-step microwave heating. M13 bacteriophage is a nanosized filamentous virus particle with a single-stranded DNA genome encapsulated by a large number of coat proteins. These amino acid rich building blocks provide a substantial amount of carbon source for the synthesis of fluorescent nanoparticles. The resulting nanoparticles from M13 bacteriophage showed good water solubility and exhibited bright blue luminescence. The selectivity and sensitivity of the phage-based nanoparticles towards Fe(iii) ions showed a quenching effect with a linear correlation and a detection limit of 8.0 μM. This process highlights the potential application of virus particles as a source for the synthesis of fluorescent carbon nanoparticles and the sensing application.

## Abstract 2

M13 bacteriophage is an attractive alternative source for fluorescent nanoparticle synthesis.

## Graphical abstract

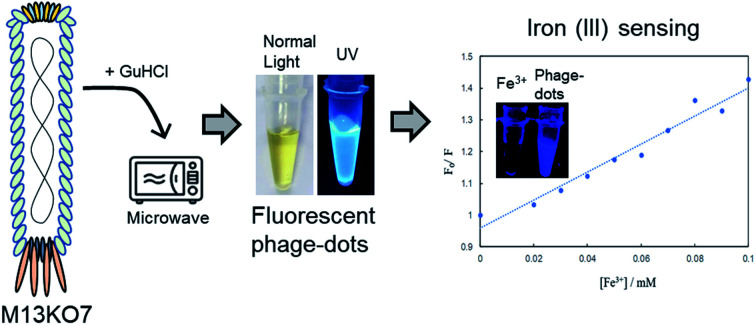

## Introduction

Nanoparticles are made up of diverse classes of nanomaterials with sizes ranging from 1 to 100 nm.^1^ Among many types of nanoparticles, fluorescent nanoparticles such as quantum dots (QDs) offer high quantum yield and photostability, which are ideal for sensors and imaging.^2,3^ However, QDs are less biocompatible because the core synthesizing materials are highly toxic heavy metals such as cadmium (Cd). Therefore, carbon-based nanoparticles generated from chemically inert carbon are emerging as an attractive alternative. They are more biocompatible, environmentally friendly and in line with the advocacy for green chemistry^4^ while still maintaining some outstanding characteristics such as high photoluminescence quantum yields, excellent photo-stability, chemical stability, storage stability, non-blinking, anti-photobleaching and good water solubility.^5,6^

Carbon nanoparticles are synthesized *via* the physical top-down route that breaks large carbon into nanometer-sized particles through processes like oxidation with strong oxidants, arc discharge,^7^ plasma treatment, and laser ablation^8^ or chemical bottom-up method which fabricates carbon nanoparticles from organic molecules through pyrolysis.^9^ Bottom-up methods through hydrothermal or microwave are more favorable due to the less complicated steps. Meanwhile, the microwave method is gaining popularity over the hydrothermal method because of the quick reaction time normally within 10 to 20 minutes and is energy efficient as compared to the hydrothermal method that requires a longer reaction time from a few hours to days under high-temperature condition. The optical performance of carbon nanoparticles is commonly improved through doping the nanoparticle surface with functional groups because heteroatoms such as nitrogen (N) on the surface could create defect levels and enhance the charge transfer, resulting in higher quantum yields.^10,11^ To introduce functional groups which provide the carbon nanoparticles with unique physiochemical characteristics such as size, quantum yield, surface charge and biocompatibility, the reactive units on the precursor plays an important role.^12^ Hence, starting materials rich in hydroxyl, carboxyl and amino groups, such as acetic acid,^13^ chitosan,^14^ citric acid,^15^ and urea^16^ are commonly used for the synthesis of carbon nanoparticles. Doping of functional groups to the carbon nanoparticles through chemical passivation involves complicated purification steps downstream.^11^ Thus, a one-pot chemical bottom-up synthesis process is more desirable.

M13 bacteriophage is a nanosized filamentous virus particle with a single-stranded DNA genome encapsulated by many coat proteins.^17^ Recently, new virus-based sensor systems using M13 bacteriophage have attracted considerable attention as functional colloidal particles.^18,19^ Some studies reported the use of bacteria^20,21^ and fungi^22,23^ as well. However, to our knowledge, the utilization of virus particles as fluorescent nanoparticles has not been reported before. Herein, we first report the use of M13 bacteriophage as a carbon source to synthesis carbon-based fluorescent nanoparticles. M13 bacteriophage used in this study is a non-enveloped filamentous virus particle that is explicit for bacteria with a vast application in the biomedical field, especially in phage display technology as a platform to present proteins or peptides.^24^ Structurally, M13 bacteriophage is a long, filamentous virus with 2700 copies of the major coat protein and several copies of the minor coat proteins, encapsulating a single-stranded DNA genome.^25^ Both the proteins and nucleic acids on M13 bacteriophages could provide a substantial amount of carbon and nitrogen for carbonization and doping. Moreover, M13 bacteriophage is harmless to human and easy to prepare in huge amounts at low cost,^26^ making it biocompatible and cost-effective for bulk production. Not to be confused with the usage of phage itself as a nanoparticle, we sought to utilize M13 bacteriophage as the carbon and nitrogen source to synthesize fluorescent carbon nanoparticles through a one-step facile microwave method.

Application of fluorescent carbon-based nanoparticles has spanned from bioimaging,^20,27,28^ drug delivery,^29–31^ to sensing.^22,23,32^ Heavy metal sensing using fluorescent carbon-based nanoparticles is achieved through a quenching mechanism in the presence of heavy metals such as silver ion (Ag^+^),^33–35^ copper ion (Cu^2+^),^36–38^ nickel ion (Ni^2+^),^36^ mercury ion (Hg^2+^),^16,37,39^ lead ion (Pb^2+^),^40–42^ or iron ion (Fe^3+^).^13,43,44^ Among these metal ion sensings, Fe^3+^ is an important element in the blood heme group and plays an essential role in oxygen metabolism, immune system, and neurotransmitter system. Excessive or deficiency in Fe^3+^ will have fatal consequences to tissues and organs.^45^ Therefore, an effective screening probe is necessary to analyze Fe^3+^ in the food, water, clinical and industrial products.^13,43,44^ In this study, fluorescent M13 bacteriophage-based nanoparticles were also evaluated for Fe^3+^ sensing application.

## Experimental

### Materials

M13KO7 was obtained from New England Biolabs, 2YT culture media was obtained from Novagen, agar–agar, citric acid, copper(ii) sulfate (CuSO_4_·5H_2_O), manganese(ii) sulfate monohydrate (MnSO_4_·H_2_O), nickel(ii) sulfate hexahydrate (NiSO_4_·6H_2_O), polyethylene glycol 6000 (PEG 6000) and potassium dihydrogen phosphate (KH_2_PO_4_), were obtained from R&M Chemicals. TG1 *Escherichia coli* cell was purchased from Agilent. Kanamycin sulfate was obtained from Amresco. Aluminium chloride hexahydrate (AlCl_3_·6H_2_O), guanidine hydrochloride (GuHCl) and zinc sulfate heptahydrate (ZnSO_4_·7H_2_O) were bought from Sigma-Aldrich, sodium chloride (NaCl) was bought from Friedemann Schmidt, iron(ii) sulfate heptahydrate (FeSO_4_·7H_2_O), potassium chloride (KCl) and sodium dihydrogen phosphate (NaH_2_PO_4_) were bought from Merck. Disodium hydrogen phosphate (Na_2_HPO_4_), iron(iii) chloride hexahydrate (FeCl_3_·6H_2_O) was purchased from Bendosen, calcium chloride (CaCl_2_) and magnesium chloride (MgCl_2_) were purchased from Fisher Scientific, chromium(iii) potassium sulfate dodecahydrate (KCr(SO_4_)_2_·12H_2_O) was purchased from QRec. All the salts were dissolved in ultrapure water purified through a Milli-Q system (18.2 MΩ cm, Millipore).

### Preparation of M13KO7 phage

A total of 10 μL of M13KO7 phage particles (10^11^) was 10-fold serial diluted and used to infect TG1 cells at OD_600nm_ 0.5 for 30 minutes at 37 °C. The infected cells were mixed with molten top agar and plated out on 2YT agar plate. The plates were incubated overnight at 37 °C to obtain plaques. To prepare helper phage, a plaque was picked to inoculate 5 mL of TG1 cells at OD_600nm_ 0.5 for 2 hours at 37 °C with shaking. Then, 500 mL of 2YT media was added and continued to culture for 1 hour before 60 μg mL^−1^ kanamycin sulfate was added. The culture was grown overnight at 30 °C with constant shaking at 600 rpm. On the next day, the cells were centrifuged down, and the M13KO7 phage particles were concentrated using PEG-precipitation. The concentrated phage particles were tittered using CFU assay^46^ and stored in phosphate-buffered saline (PBS) at 4 °C until use.

### Synthesis of phage-based nanoparticles

Phage-based nanoparticles (P-NPs) were synthesized through a one-step hydrothermal carbonization method. A total of 5 mL M13KO7 phage particles (10^12^ pfu mL^−1^) was stirred with 6 M GuHCl in a final volume of 7 mL for 3 hours at room temperature to denature the phage proteins. Subsequently, P-NPs were synthesized using the microwave. The mixture was heated at 700 W in a microwave pressure cooker for 5 minutes or until the solution was completely dried. After the reaction was cooled down, the dark brown product was re-dissolved with 10 mL of ultrapure water and centrifuged at 8000 rpm for 30 minutes to remove large aggregates. The resulting supernatant was clarified through a 0.22 μM filter (Sartorius, Germany) followed by separation using a 3K centrifugal device (Pall, USA).

### Characterization

The size and morphology of the P-NPs were characterized using transmission electron microscopy (TEM) (a JEOL JEM2100 microscope operated at 200 kV), atomic force microscopy (AFM) (Seiko SPI-3700/SPA-300, Seiko Instruments Inc., Japan). UV-vis (absorption) spectra were recorded using a UV-vis-NIR spectrophotometer (V670, JASCO, Japan) and NanoDrop™ 2000c spectrophotometer (Thermo Scientific, United States). The quantum yield of fluorescence of the P-NPs (excitation at 315 nm and emission at 365 nm) was determined by the common reference-based method using quinine sulfate as the standard. Fluorescence excitation and emission wavelengths were obtained using a fluorescent spectrophotometer (Varian Cary Eclipse, Agilent, United States) where the excitation and emission slits were both set at 10 nm and samples (3 mL final volume) were measured in cuvettes with a 10 mm optical path. Fourier transform infrared (FT-IR) spectra were measured on the FT-IR spectrophotometer (FTIR 4200 spectrometer, JASCO, Japan). X-ray photoelectron spectroscopy (XPS) data of the dried sample was measured by ESCALAB 250 XPS system having an Al Kα X-ray source (Thermo Scientific, United States) for determining the composition and chemical bonding configurations of P-NPs. The time decay analysis of fluorescence was carried out by a C11367 Quantaurus-Tau compact fluorescence lifetime spectrometer (Hamamatsu Photonics K.K., Japan).

### Sensitivity of phage-based nanoparticles to pH

P-NPs stock solution was diluted 1 : 300 in phosphate buffer with the pH adjusted to a range from 1 to 12. The mixture was incubated at room temperature for 1 hour and the fluorescence intensity was measured at 320/380 nm (excitation/emission).

### Selectivity and sensitivity of phage-based nanoparticles to Fe^3+^

P-NPs stock solution was diluted 1 : 300 in citrate-phosphate buffer, pH 3. The selectivity of P-NPs to Fe^3+^ was tested by adding different metal ions solution (K^+^, Na^+^, Ca^2+^, Mg^2+^, Mn^2+^, Ni^2+^, Zn^2+^, Cu^2+^, Al^3+^, Cr^3+^, Fe^2+^ and Fe^3+^) in a final concentration of 2 mM to the diluted P-NPs solution. Sensitivity of P-NPs to Fe^3+^ was examined similarly by adding Fe^3+^ solution in a final concentration of 0.01 mM to 1.50 mM to the diluted P-NPs solution. All the metal solutions was dissolved and diluted in citrate–phosphate buffer, pH 3. The mixture was incubated at room temperature for 2 minutes and the absorbance and fluorescence intensity was measured. Fluorescence intensity was recorded at 320/380 nm (excitation/emission).

## Results and discussion

### Preparation and characterization of phage-based nanoparticles (P-NPs)

**Scheme 1:**
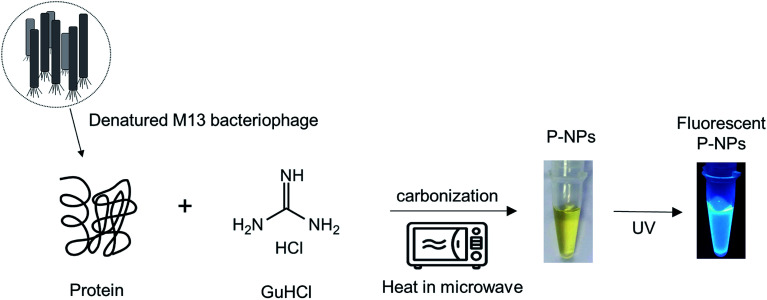
Schematic representation of the synthesis of P-NPs from M13 bacteriophage through a one-step microwave hydrothermal method.

Numerous biomolecules have been previously utilized to produce fluorescent nanoparticles.^47^ In this work, M13 bacteriophage particles were utilized for the first time as the raw material to synthesize fluorescent nanoparticles. The water-soluble phage-based nanoparticles (P-NPs) were synthesized through a one-step microwave hydrothermal method as illustrated in Scheme 1. GuHCl was functioned as protein denaturant to denature the M13 bacteriophage and release the phage proteins^48^ which served as the carbon source for carbonization while at the same time GuHCl provided nitrogen (N) for N-doping.^49^ GuHCl at 6 M concentration is usually used for the denaturation and complete dissociation of protein structures. We hypothesized that the amino acid/protein-rich building blocks of M13 bacteriophage would provide substantial amount of carbon source for the synthesis of fluorescent nanoparticles, since various natural carbon-rich materials including amino acid/protein such as plant extracts,^50–52^ sugars,^29,53,54^ proteins,^55–57^ amino acids^32,58,59^ have been widely utilized to generate fluorescent carbon nanoparticles.

**Fig. 1:**
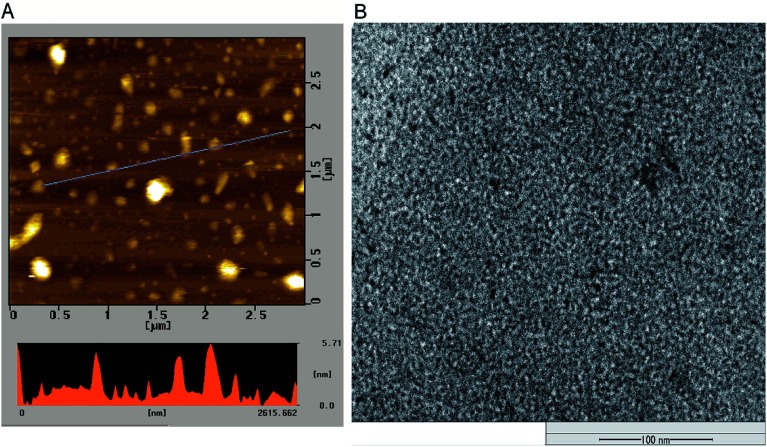
(A) AFM image of P-NPs and (B) TEM image of P-NPs.

The morphology and size of P-NPs were characterized using AFM and TEM. As shown in Fig. 1A, the diameter of P-NPs ranged from 40 nm to 70 nm and were dispersed from one another in AFM image. P-NPs have a size in the range of 100 nm that allowed it to be classified as nanoparticles.^1^ The TEM image (Fig. 1B) showed many black spots of P-NPs, but the aggregation of P-NPs cannot be avoided in the drying process for AFM observation.

**Fig. 2:**
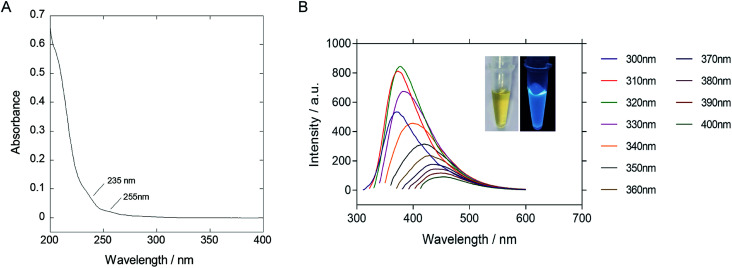
(A) UV-vis absorbance spectrum of P-NPs. (B) Fluorescence emission spectrum of P-NPs obtained under excitation at wavelength ranged from 300 to 400 nm. P-NPs shown maximum emission at 380 nm when excited at 320 nm.

The as-synthesized P-NPs demonstrated good water-solubility. The resulting solution was yellowish and transparent under visible light but emitting strong blue photoluminescence under UV illumination (Fig. 2B inset). The UV-vis absorption spectrum (Fig. 2A) showed a peak at 205 nm, corresponded to the π–π* transition of the carbon backbone and carbonyl group. Shoulder peaks at 235 nm and 255 nm represent the n–π* transition of a carbonyl group. The fluorescence emission spectrum (Fig. 2B) was plotted against a series of excitation wavelengths ranging from 300 nm to 400 nm and demonstrated an excitation-dependent fluorescence behavior. The emission peak gave a right-handed shift when the excitation wavelength increased. The excitation dependent fluorescence in P-NPs suggests the distribution of different emissive sites on the P-NPs, as similar to the case of other fluorescence carbon dots reported before.^60^ However, the origin of different emissive sites is not clear because of the heterogeneous components of P-NPs.

The highest emission intensity was observed at 380 nm when the P-NPs solution was excited at 320 nm. Therefore, the fluorescence intensity of the following experiments was measured using an excitation and emission wavelength of 320 nm and 380 nm respectively. The quantum yield (QY) was evaluated to be 14.8% using quinine sulfate as the standard. The high QY in P-NPs could be linked to the N-doping effect from the nitrogen atom present in M13 bacteriophage's protein and GuHCl, as reported in previous studies where N-doped carbon nanoparticles demonstrated high QY.^10,11^

**Fig. 3:**
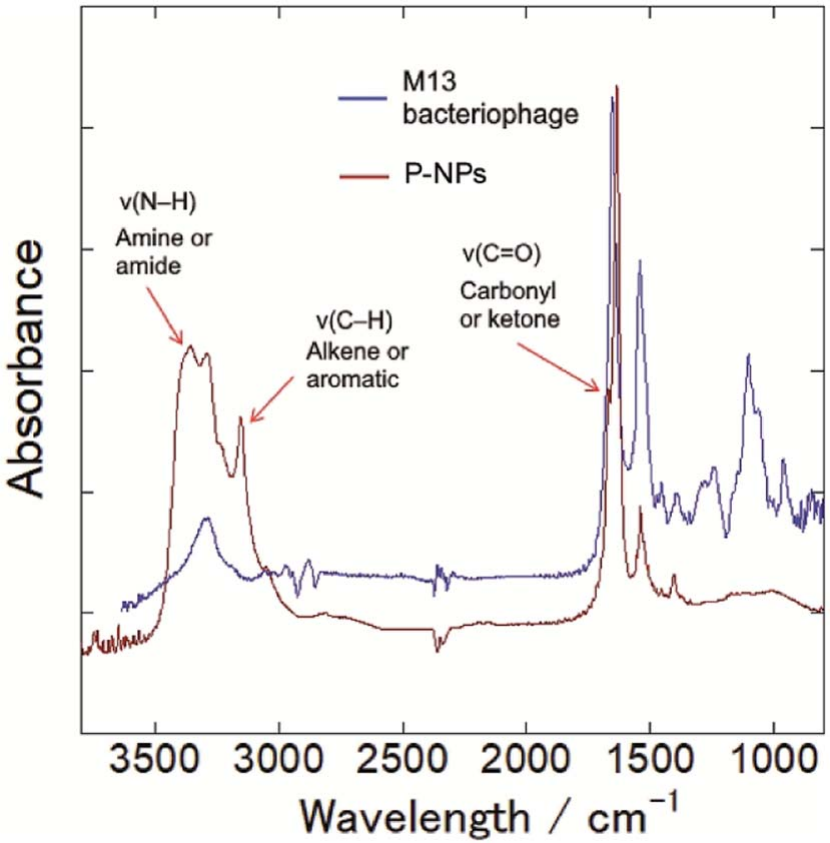
FT-IR spectra of M13 bacteriophage (blue line) and as-synthesized P-NPs (red line), indicating amine, amide, alkene, aromatic and ketone groups in P-NPs.

The functional groups on P-NPs’ surface were analyzed through Fourier transform infrared (FT-IR) spectroscopy. Fig. 3 showed the IR spectra that highlighted the functional groups existing on the P-NPs. The absorption band ranging from 3300–3500 cm^−1^ is attributed to the stretching vibration of N–H (*ν*(N–H)), which may originate from primary amine and amide groups. The peak at 3000–3100 cm^−1^ is attributed to the stretching vibration of C–H (*ν*(C–H)), which may originate from either the alkene (=C–H) or aromatic (C–H) groups. A sharp peak at 1600–1700 cm^−1^ can be contributed by the stretching vibration of C=O (*ν*(C=O)), which may come from the carbonyl and ketone groups. A peak at 1550 cm^−1^ can be attributed to the bending vibration of N–H (*δ*(N–H)). The IR absorbance analysis suggested the presence of amine, amide, alkenes, aromatic, carbonyl and ketone groups in the P-NPs. The reason P-NPs are rich in amino and amide functional groups could be attributed to the abundance of amino acids, which are the building blocks of the precursor M13 bacteriophage coat proteins.^25^ As seen in the IR spectra, M13 bacteriophage has peaks observed at 3300 cm^−1^, 1700 cm^−1^ and 1550 cm^−1^ which could correspond to the stretching vibration of N–H, C=O and bending vibration of N–H respectively. The spectra suggested the M13 bacteriophage has amine, amide and carbonyl groups, which are the functional groups of amino acids. The carbonyl-rich condition also allowed for the good water solubility property in as-synthesized P-NPs.

**Fig. 4:**
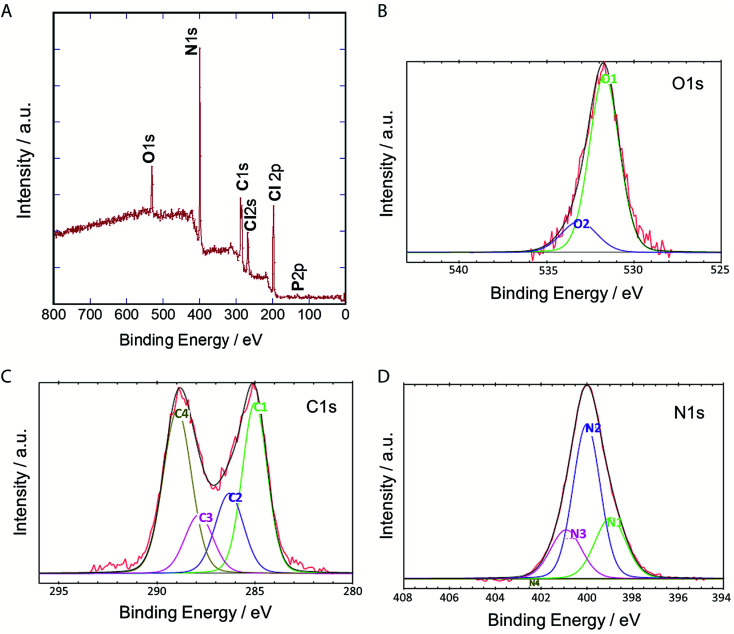
(A) XPS spectrum of P-NPs and de-convoluted spectra of (B) O 1s (C) C 1s and (D) N 1s.

To clarify their elemental composition and chemical bonding nature, we examined the X-ray photoelectron (XPS) spectrum of P-NPs. The XPS survey spectrum (Fig. 4A) showed 5 peaks, indicating that P-NPs are composed of carbon (C), nitrogen (N), oxygen (O), chlorine (Cl), and phosphorus (P) with percentages of 40.2% (C), 42% (N), 7.1% (O), 10.2% (Cl), and 0.5% (P) respectively. These are consistent with the elements on amino acid, nucleic acid, and guanidine hydrochloride. The high-resolution XPS spectra in Fig. 4B–D and their peaks were de-convoluted with several chemical bonding states according to the literature.^61^ The O 1s peak was de-convoluted with binding energies of approximately 531.7 eV (O1) and 533.2 eV (O2) (Fig. 4B), which are assigned to C=O for O1 (82.7%) and C–O groups for O2 (13.7%), indicating the rich C=O group of P-NPs. The high-resolution C 1s peak was de-convoluted with binding energies of approximately 285.0 eV (C1), 286.3 eV (C2), 287.9 eV (C3) and 289.0 eV (C4) (Fig. 4C), which were attributed to the following bonding and content (%): C–C, C=C and C–H for C1 (33.3%), C–N, C=N, and C–O for C2 (17.7%), C=O for C3 (12.9%) and O=C–O, N=CN_2_ for C4 (36.0%), indicating the high content of C–N bonding in the P-NPs. The N=CN_2_ group for C4 may originate from guanidine at the synthesis of P-NPs. This indicates the incorporation of guanidine into the P-NPs at the synthesis level. The N 1s spectrum of P-NPs shows 3 peaks that were de-convoluted with binding energies of ∼399.0 eV (N1), 400.0 eV (N2), and 400.9 eV (N3) (Fig. 4D), which were attributed to the following groups and content (%): pyridinic N for N1 (24.9%), amino N and pyrrolic N for N2 (55.2%), graphitic N for N3 (19.9%). The high content of amino/pyrrolic N and the low content of graphitic N suggest small graphitic structures in the P-NPs. This XPS data is well-matched with FT-IR analysis.

### Fluorescence quenching at different pH

**Fig. 5:**
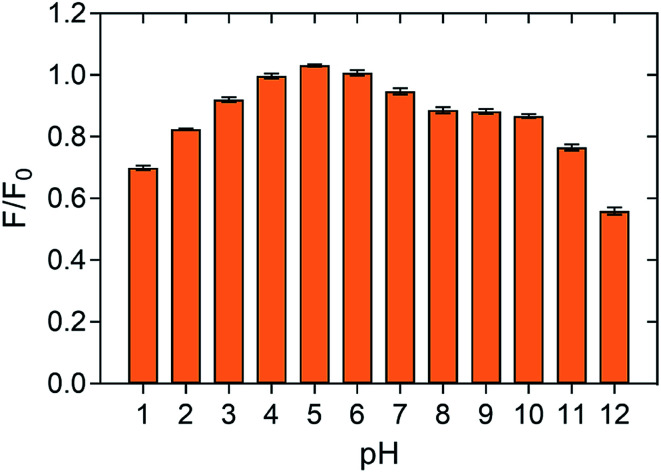
Normalized fluorescence intensity of P-NPs at different pH. P-NPs were quenched at extreme pH and exhibited maximum fluorescence intensity at pH 3–7. The error bars indicate the standard deviations of three independent measurements.

The change of fluorescence emission intensity of as-prepared P-NPs in different pH was measured to investigate the stability of P-NPs over a wide range of pH. The fluorescence intensity after treatment in different pH solutions is compared with the fluorescence intensity of P-NPs in ultrapure water. As demonstrated in Fig. 5, P-NPs showed maximum fluorescence intensity at pH 3–7. The sensitivity of the P-NPs was slightly dependent on pH as the fluorescence intensity dropped at extremely acidic (pH 1) and extremely basic solutions (pH 11–12). The quenching effect of pH could be ascribed to the protonation of amine groups under strong acidic solution and deprotonation of the carboxylic group on the P-NPs under strong alkaline environment.^62^ Overall, P-NPs exhibited strong fluorescence intensity over the pH ranged from 2 to 10, suggesting the as-prepared P-NPs are stable and sensitive to act as a sensor for Fe^3+^ ions that is acidic in aqueous form. As Fe^3+^ precipitates at pH 3.5 and above, pH 3 was therefore adopted as the optimum pH to determine the quenching effect of Fe^3+^ in this study.

### Selectivity of P-NPs to Fe^3+^

**Fig. 6:**
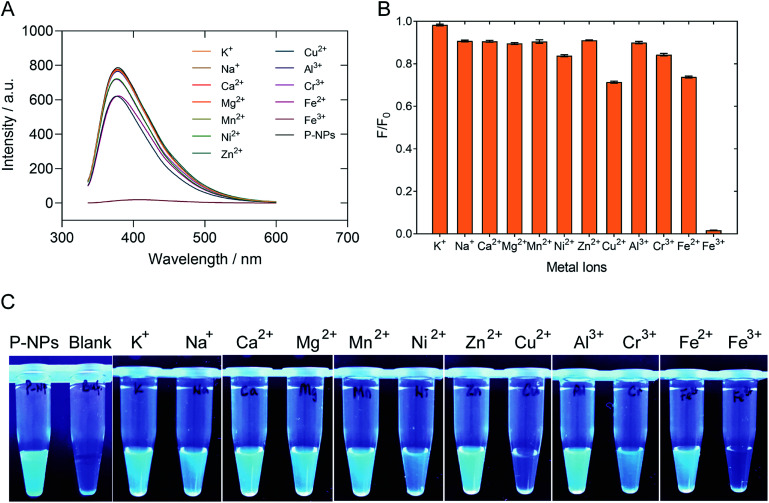
(A) Spectrum of fluorescence quenching upon addition of different metal ions at 2 mM concentration. P-NPs quenched completely when Fe^3+^ was added, quenched slightly in the presence of Cu^2+^, Cr^3+^ and Fe^2+^ but remained relatively inert when other metal ions were added. (B) Normalized fluorescence intensity of P-NPs upon the addition of different metal ions. The error bars indicate the standard deviations of three independent measurements. (C) Observation of fluorescence intensity upon addition of different metal (from left: P-NPs, blank buffer solution, K^+^, Na^+^, Ca^2+^, Mg^2+^, Mn^2+^, Ni^2+^, Zn^2+^, Cu^2+^, Al^3+^, Cr^3+^, Fe^2+^, Fe^3+^) under UV light.

The selectivity of P-NPs towards Fe^3+^ was examined by using various metal ions, such as K^+^, Na^+^, Ca^2+^, Mg^2+^, Mn^2+^, Ni^2+^, Zn^2+^, Cu^2+^, Al^3+^, Cr^3+^, Fe^2+^ and Fe^3+^ at 2 mM concentration. The changes in fluorescence intensity were recorded and demonstrated in Fig. 6. The fluorescence intensity of P-NPs was quenched completely when Fe^3+^ was added but remained constant with no significant quenching effect when other metal ions were added. Fig. 6C showed the P-NPs under UV illumination after the addition of various metal ions. The result indicates selective detection of the P-NPs towards Fe^3+^ whilst remain relatively inert to other metal ions at 2 mM concentration.

### Sensitivity of P-NPs to Fe^3+^

**Fig. 7:**
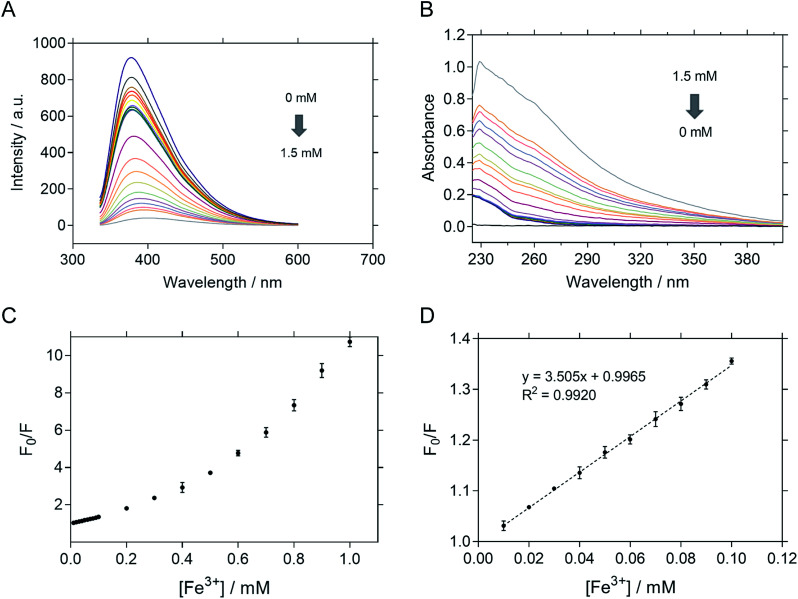
(A) Spectrum of fluorescent quenching upon addition of different concentrations of Fe^3+^. The fluorescent intensity decreased gradually when the concentration of Fe^3+^ increase. (B) Absorbance upon addition of different concentrations of Fe^3+^. (C) Sensitivity of P-NPs towards Fe^3+^ with concentration gradient from 0.1 mM to 1 mM. The error bars indicate the standard deviations of three independent measurements. (D) Stern–Volmer plot of P-NPs toward different concentrations of Fe^3+^. The plot shown good linear correlation (*R*^2^ = 0.9920) over the range of 0.01 mM to 0.1 mM.

The sensitivity of P-NPs in Fe^3+^ detection was examined by adding Fe^3+^ in a series of concentrations ranging from 10 μM to 100 μM. There was an inversely proportional relationship between the fluorescence intensity and Fe^3+^ concentration. The fluorescence intensity of P-NPs decreased gradually (Fig. 7A) whereas the absorbance increased gradually (Fig. 7B) when the concentration of Fe^3+^ was increased, indicating the ability of P-NPs to quantitatively detect the presence of Fe^3+^. The sensitivity of the P-NPs towards Fe^3+^ was demonstrated in Fig. 7C. Stern–Volmer plot was used as the calibration plot (Fig. 7D). The plot demonstrated good linear correlation (*R*^2^ = 0.9920) between *F*_0_/*F* and concentration of Fe^3+^ over the range of 10 μM to 100 μM, where *F*_0_ and *F* represent the fluorescence intensity in the absence and presence of Fe^3+^ respectively. The limit of detection (LOD) was then calculated using 3*S*_*yx*_/slope, where *S*_*yx*_ represents the standard deviation of the residuals of calibration samples and slope represents the slope of the calibration plot.^63^ The LOD of P-NPs to Fe^3+^ was determined to be 8.0 μM. Table 1 compares the LOD of carbon-dots derived from other carbon sources towards Fe^3+^ that have been reported previously. Although the sensitivity of the synthesized P-NPs was relatively high compared to other carbon-dots, this does not discount the possibility of bacteriophages to be used as an alternative source for fluorescent nanoparticles synthesis.

**Table 1:** Linear range and limit of detection towards Fe^3+^ of various carbon dots synthesized from different materials using microwave synthesis method

Precursors	Quantum yield (%)	Linear range (μM)	Limit of detection (μM)	Ref.
Banana peels	—	2–16	0.21	64
Goose feather	17.1	2–7	0.20	65
*o*-Phenylenediamine	38.5	0–100	0.02	66
*Bauhinia flower*	27.00	10–350	0.01	67
dl-Malic acid	15.13	6–200	0.80	68
l-glutamic acid	12.45	8–80	3.80	69
M13 bacteriophage	14.80	10–100	8.00	This work

### Fluorescence quenching mechanism of P-NPs to Fe^3+^

**Fig. 8:**
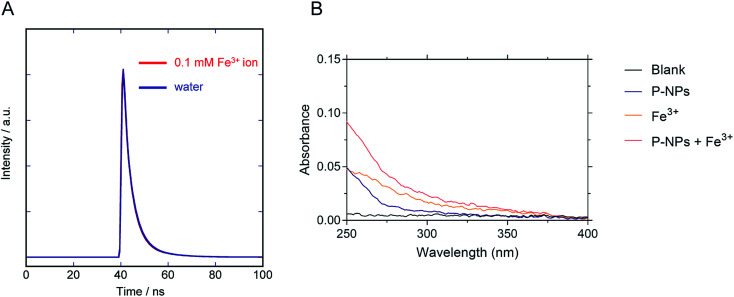
(A) Fluorescence decay curve of the P-NPs in the presence and the absence of 0.1 mM Fe^3+^. (B) Absorbance of blank buffer, P-NPs, 0.1 mM Fe^3+^, and mixture of P-NPs and 0.1 mM Fe^3+^.

We observed the reduction in the fluorescence intensity (*i.e.* quenching) of P-NPs in the presence of Fe^3+^. There are two possibilities for this quenching process: (i) electron/energy transfer from P-NPs to Fe^3+^ and (ii) inner filter effect (IFE) by Fe^3+^.^70^ In the case of the former, the fluorescence intensity and the fluorescence lifetime reduced in the presence of quencher (Fe^3+^). In the latter case, there should be no change of the fluorescence lifetime and spectral overlapping between the excitation of P-NPs and the absorption of Fe^3+^. Hence, to ascertain the main reason for quenching, the lifetime decay of P-NP was collected in the absence and presence of 0.1 mM Fe^3+^. It is evident from Fig. 8A that the average fluorescence decay 〈*τ*〉 of the P-NPs was almost identical in the presence (4.6 ns) and the absence (4.7 ns). Besides, the UV absorption of Fe^3+^ (Fig. 8B) showed an overlap to the excitation (320 nm) of P-NPs. Thus, we consider that fluorescence quenching of P-NPs by Fe^3+^ occurred primarily *via* the IFE mechanism in the quenching process.

## Conclusions

In brief, M13 bacteriophage particles have been successfully utilized as a carbon source to synthesis water-soluble phage-based nanoparticles through simple and fast microwave heating. The resulting nanoparticles showed fluorescence intensity with peak emission at 380 nm when excited at 320 nm. It is also highly stable under moderate pH conditions and demonstrated a quenchiometric detection of Fe^3+^*via* fluorescence quenching, which may be caused by inner filter effect (IFE) by Fe^3+^. This reflects the possibility of utilizing virus particles as a carbon source for nanoparticle synthesis.

## Conflicts of interest

There are no conflicts to declare.

## Supplementary Material
